# Magnetoimpedance in Symmetric and Non-Symmetric Nanostructured Multilayers: A Theoretical Study

**DOI:** 10.3390/s19081761

**Published:** 2019-04-12

**Authors:** Nikita A. Buznikov, Galina V. Kurlyandskaya

**Affiliations:** 1 Scientific and Research Institute of Natural Gases and Gas Technologies—Gazprom VNIIGAZ, Razvilka, Leninsky District, Moscow Region 142717, Russia; n_buznikov@mail.ru; 2 Department of Magnetism and Magnetic Nanomaterials, Institute of Natural Sciences and Mathematics, Ural Federal University, Ekaterinburg 620002, Russia; 3 Department of Electricity and Electronics, Basque Country University UPV/EHU, 48940 Leioa, Spain

**Keywords:** magnetic multilayers, magnetoimpedance, modeling, magnetic sensors, magnetic biosensors

## Abstract

Intensive studies of the magnetoimpedance (MI) effect in nanostructured multilayers provide a good phenomenological basis and theoretical description for the symmetric case when top and bottom layers of ferromagnet/conductor/ferromagnet structure have the same thickness and consist of one magnetic layer each. At the same time, there is no model to describe the MI response in multilayered films. Here, we propose the corresponding model and analyze the influence of the multilayer parameters on the field and frequency dependences of the MI. The approach is based on the calculation of the field distribution within the multilayer by means of a solution of lineralizied Maxwell equations together with the Landau–Lifshitz equation for the magnetization motion. The theoretical model developed allows one to explain qualitatively the main features of the MI effect in multilayers and could be useful for optimization of the film parameters. It might also be useful as a model case for the development of MI magnetic biosensors for magnetic biomarker detection.

## 1. Introduction

The magnetoimpedance (MI) effect implies a strong dependence of the complex impedance of a ferromagnetic conductor on an external magnetic field [1,2]. Since its rediscovery [3,4,5,6], the effect has attracted much attention due to its remarkable advantages for the development of high-sensitive magnetic field detectors [7,8]. The origin of MI can be explained in the framework of the classical skin effect, i.e., the tendency of an alternating electric current to be distributed within a conductor in such a way that the current density is largest near the conductor surface. The MI is related to the changes in the skin depth with the permeability of the ferromagnetic conductor and is observed in soft magnetic materials, which exhibit variation in permeability at low external magnetic fields. The effect was studied in detail in different magnetic materials, in particular, in amorphous wires and ribbons, electroplated wires, glass-coated microwires, and thin-film based systems.

**Figure 1:**
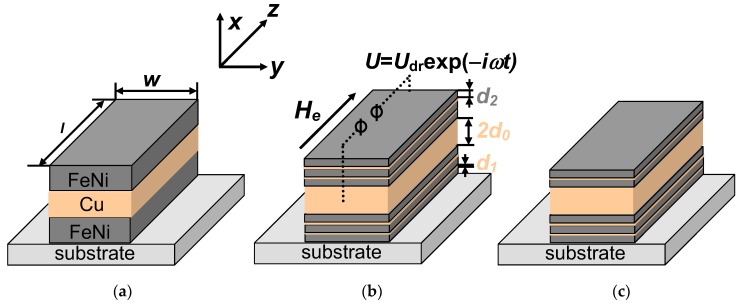
Schematic representation of the MI multilayered sensitive elements. (**a**) Classic “MI sandwich” without nanostructuring of magnetic layers. (**b**) MI symmetric multilayer with the same total thickness of FeNi top and bottom layers, both deposited as multilayers with Cu spacers. (**c**) MI non-symmetric multilayer with different total thicknesses of top and bottom layers: top multilayer contains two FeNi sub-layers and bottom multilayer contains three FeNi sub-layers in this particular case.

Maximum magnitudes of the impedance variation and field sensitivity were obtained in Co-based amorphous wires and glass-coated microwires. However, for sensor miniaturization, thin-film structures could be more attractive materials. Experimental studies of the MI effect have been performed in films with different structures, such as single-layer ferromagnetic films [9,10] and three-layered films called “MI sandwich” [10,11,12,13,14] (see Figure 1a). Changes in the film impedance with the external magnetic field become high when the skin penetration depth reaches the order of the film thickness. For a soft magnetic film with a thickness of 1μm, this condition is valid within the gigahertz frequency range [12]. To observe large changes in the impedance at moderate frequencies, three-layered film structures consisting of two soft magnetic films separated by a non-magnetic layer were proposed, designed, tested, and described by appropriate models [12,14]. Typical material for soft magnetic layers is nanostructured permalloy, and highly conductive Cu, Al, or Au are used for the central layer material. Thin-film sensitive elements with thicknesses of the order of microns are required for many sensor applications, including MI [13,15].

High field sensitivity of the impedance in film structures can be obtained when permalloy layers have low coercivity, high permeability, and well defined in-plane magnetic anisotropy with low local anisotropy axes distribution. However, the out-of-plane component of the anisotropy can appear in permalloy films, when the film thickness increases above the critical thickness of the transition into a “transcritical state” [16,17,18]. The value of the thickness corresponding to the transition depends on many parameters, including the working gas pressure during sputtering deposition [17]. This value can vary in the range from a few nanometers to a few hundred nanometers [17,19]. The appearance of the out-of-plane component of the anisotropy is ascribed to columnar structure formation, magnetocrystalline, and/or magnetoelastic anisotropy [19]. As a result, degradation of the soft magnetic properties takes place due to the transition into the “transcritical state” [16,19]. To avoid the “transcritical state” transition and to increase the total thickness of soft magnetic layers, nanostructured multilayers have been proposed and developed [20,21].

The influence of different parameters, such as the thickness of magnetic layers, material, and thickness of non-magnetic interfaces, on the magnetic properties and the MI response in multilayers was studied experimentally [22,23,24,25,26,27,28]. It was also demonstrated that MI in nanostructured multilayered films could be promising for the development of magnetic biosensors [29,30]. In a magnetic biosensor, non-uniform magnetic fields having a complex configuration should be analyzed. In this connection, recently, non-symmetric nanostructured multilayered films have attracted considerable attention [31,32]. Non-symmetric films were obtained by the deposition of top and bottom ferromagnetic parts of a multilayer with different thicknesses. It was found that the symmetric multilayered films have the highest field sensitivity. At the same time, non-symmetric multilayers allow one to obtain a higher MI response at high frequencies [32].

Although the MI effect in nanostructured multilayers was intensively studied in experiments, to the best of our knowledge, there is no model to describe the MI response in multilayered films. In this paper, we propose the corresponding model and analyze the influence of the multilayer parameters on the field and frequency dependences of the MI. The approach is based on the calculation of the field distribution within the multilayered film by means of a simultaneous solution of lineralizied Maxwell equations and the Landau–Lifshitz equation for the magnetization motion. Both the symmetric and non-symmetric nanostructured multilayered films are studied. The model developed allows one to explain qualitatively the main features of the MI effect in multilayers and could be useful for optimization of the film parameters.

## 2. Model

Let us consider a film structure, [F/X]_m_/F/C/[F/X]_n_/F, shown schematically in Figure 1. The structure consists of a highly conductive non-magnetic central layer, C, of a thickness, 2*d*_0_, and two external (top and bottom) multilayers. The external multilayers contain soft magnetic layers, F, of a thickness, *d*_2_, separated by non-magnetic layers (spacers), X, of a thickness, *d*_1_. The corresponding conductivities of the layers, C, X, and F, are *σ*_0_, *σ*_1_, and *σ*_2_. Note that top and bottom multilayers may have different thicknesses, *m* ≠ *n*. It is taken into account that materials of the central layer, C, and spacers, X, may be different [27,33]. The film structure length and width are *l* and *w*, respectively.

It is further supposed that all magnetic layers have the same physical properties. Usually, during the deposition of multilayered films, a constant magnetic field is applied along the short side of the film in order to induce the transverse magnetic anisotropy. To take into account this fact, we assume that the magnetic layers have uniaxial in-plane magnetic anisotropy, and the angle, *ψ*, of deviation of the anisotropy axis from the transverse direction is relatively small.

We also assume that the value of the permeability in the ferromagnetic layers is governed by the magnetization rotation only. This approximation is valid at sufficiently high frequencies (above 10 MHz), when the domain-wall motion is damped [34,35]. Furthermore, due to the averaging over the domain structure, the permeability tensor has a quasi-diagonal form. In this case, the MI of the multilayered film depends on the value of the transverse permeability only.

The alternating driving voltage, *U* = *U*_dr_exp(−*iωt*) (where *ω* is the angular frequency, *t* is the time, and *i* is the imaginary unit), is applied to the multilayered structure, and the external magnetic field, *H_e_*, is parallel to the long side of the film (see Figure 1b).

Let us restrict our consideration by the case of not too high frequencies when *ωl*/*c* << 1, where *c* is the speed of light in vacuum. Then, the field distribution in the film can be considered to be independent of the coordinate, *z*. For the film length, *l* = 1 cm, this approximation is valid at frequencies, *f* = *ω*/2*π* << 5 GHz. Moreover, since the film width, *w*, is much higher than its thickness, neglecting edge effects, we can suppose that the electromagnetic fields depend only on the coordinate, *x*, perpendicular to the film plane. This approach is adequate when the film width exceeds some critical value. This critical value, *λ*, depends on the layer thicknesses and static permeability in the magnetic layers [36,37,38]. Estimations show that *λ* ≈ 10 μm for typical parameters of the multilayered films studied below.

In the one-dimensional approximation, the amplitudes of the longitudinal electric field, *e*_0_, and the transverse magnetic field, *h*_0_, in the central non-magnetic layer, C, −*d*_0_ < *x* < *d*_0_, satisfy Maxwell equations centimeter-gram-second (cgs) system of units is used:

(1) $$\begin{array}{l} -\frac{de_{0}}{dx}=(i\omega /c)h_{0}\;,\\ \frac{dh_{0}}{dx}=(4\pi \sigma_{0}/c)e_{0}\;. \end{array}$$

The solution of Equation (1) can be expressed as follows:

(2) $$\begin{array}{l} e_{0}=(cp_{0}/4\pi \sigma_{0})[A_{0}\cosh(p_{0}x)+B_{0}\sinh(p_{0}x)]\;,\\ h_{0}=A_{0}\sinh(p_{0}x)+B_{0}\cosh(p_{0}x)\;. \end{array}$$

where, *A*_0_ and *B*_0_ are the constants, *p*_0_ = (1 − *i*)/*δ*_0_ and *δ*_0_ = *c*/(2*πωσ*_0_)^1/2^. Note that for the symmetric film, *m* = *n*, the constant, *B*_0_, is equal to zero.

The solution of the Maxwell equations for the field amplitudes in the non-magnetic spacers, X, can be presented in the following form:

(3) $$\begin{array}{l} e_{1}^{\left(j\right)}=(cp_{1}/4\pi \sigma_{1})[A_{1}^{\left(j\right)}\cosh(p_{1}x)+B_{1}^{\left(j\right)}\sinh(p_{1}x)]\;,\\ h_{1}^{\left(j\right)}=A_{1}^{\left(j\right)}\sinh(p_{1}x)+B_{1}^{\left(j\right)}\cosh(p_{1}x)\;. \end{array}$$

where, $e_{1}^{\left(j\right)}$ and $h_{1}^{\left(j\right)}$ are the amplitudes of the longitudinal electric field and the transverse magnetic field, respectively; *j* = 1, …, *m* + *n* is the non-magnetic layer number; $A_{1}^{\left(j\right)}$ and $B_{1}^{\left(j\right)}$ are the constants; *p*_1_ = (1 − *i*)/*δ*_1_ and *δ*_1_ = *c*/(2*πωσ*_1_)^1/2^.

The field amplitudes, $e_{2}^{\left(k\right)}$ and $h_{2}^{\left(k\right)}$, in the magnetic layers, F, are given by:

(4) $$\begin{array}{l} e_{2}^{\left(k\right)}=(cp_{2}/4\pi \sigma_{2})[A_{2}^{\left(k\right)}\cosh(p_{2}x)+B_{2}^{\left(k\right)}\sinh(p_{2}x)]\;,\\ h_{2}^{\left(k\right)}=A_{2}^{\left(k\right)}\sinh(p_{2}x)+B_{2}^{\left(k\right)}\cosh(p_{2}x)\;. \end{array}$$

where, *k* = 1, …, *m* + *n* + 2 is the magnetic layer number; $A_{2}^{\left(k\right)}$ and $B_{2}^{\left(k\right)}$ are the constants; *p*_2_ = (1 − *i*)/*δ*_2_, *δ*_2_ = *c*/(2*πωμσ*_2_)^1/2^ and *μ* is the transverse permeability.

To find the transverse permeability in the magnetic layers, we neglect the contribution of the exchange energy. More rigorous theoretical treatment requires the inclusion of the exchange-conductivity effect in the model [39,40]. However, the contribution of the exchange-conductivity effect to the MI response is relatively low within the high-frequency range. The solution of the linearized Landau–Lifshitz equation results in the following expression for the transverse permeability, *μ*, in the ferromagnetic layers [41]:

(5) $$\mu =1+\frac{\gamma 4\pi M(\gamma 4\pi M+\omega_{1}-i\kappa \omega )\sin^{2}\theta }{(\gamma 4\pi M+\omega_{1}-i\kappa \omega )(\omega_{2}-i\kappa \omega )-\omega^{2}}\;,$$

(6) $$\begin{array}{l} \omega_{1}=\gamma [H_{a}\cos^{2}(\theta -\psi )+H_{e}\sin\theta ]\;,\\ \omega_{2}=\gamma [H_{a}\cos\{2(\theta -\psi )\}+H_{e}\sin\theta ]\;. \end{array}$$

where *M* is the saturation magnetization of the magnetic layers, *γ* is the gyromagnetic constant, *κ* is the Gilbert damping parameter, *θ* is the equilibrium magnetization angle, and *H_a_* is the anisotropy field in the ferromagnetic layers.

The equilibrium magnetization angle, *θ*, can be found by minimizing the free energy. The free energy can be presented as a sum of the uniaxial anisotropy energy and Zeeman energy. The minimization procedure results in the following equation for the magnetization equilibrium angle, *θ*:

(7) $$H_{a}\sin(\theta -\psi )\cos(\theta -\psi )=H_{e}\cos\theta \;.$$

To describe the field distribution in the external regions, we use the approximate solution for the vector potential obtained previously [37,42] in the case, *d* << *w*, where *d* = 2*d*_0_ + (*m* + *n*)*d*_1_ + (*m* + *n* + 2)*d*_2_ is the total multilayer thickness. The field amplitudes are given by:

(8) $$\begin{array}{l} e_{s}=C_{s}\frac{i\omega }{c}\left[\frac{l}{2w}\log\left(\frac{R+w}{R-w}\right)-\frac{2x}{w}\arctan\left(\frac{wl}{2Rx}\right)+\frac{1}{2}\log\left(\frac{R+l}{R-l}\right)\right]\;,\\ h_{s}=-C_{s}\frac{4lx}{R}\left[\frac{R^{2}+4x^{2}}{4R^{2}x^{2}+l^{2}w^{2}}-\frac{1}{R^{2}-w^{2}}-\frac{1}{R^{2}-l^{2}}\right]+C_{s}\frac{2}{w}\arctan\left(\frac{wl}{2Rx}\right)\;. \end{array}$$

where the subscripts, *s* = 3 and *s* = 4, correspond to the bottom and top external region, respectively, *C_s_* are the constants, and *R* = (*l*^2^ + *w*^2^ + 4*x*^2^)^1/2^. In the symmetric film, *m* = *n*, the distribution of the electric field in the external region is symmetrical with respect the multilayer center, and *C*_3_ = *C*_4_.

Thus, the field distribution within the 2(*m* + *n*) + 3 layers of the film is described by Equations (2) to (4). The total number of constants in Equations (2) to (4) is equal to 4(*m* + *n*) + 6. The constants, *A*_0_, *B*_0_, $A_{1}^{\left(j\right)}$, $B_{1}^{\left(j\right)}$, $A_{2}^{\left(k\right)}$, and $B_{2}^{\left(k\right)}$, can be found from the continuity conditions for the amplitudes of the electric and magnetic fields at the interfaces between different layers. In addition, we should take into account that the driving voltage with the amplitude, *U*_dr_, is applied to the film region, −*t*_1_ < *x* < *t*_2_, where *t*_1_ = *d*_0_ + *nd*_1_ + (*n* + 1)*d*_2_ and *t*_2_ = *d*_0_ + *md*_1_ + (*m* + 1)*d*_2_. Then, the boundary conditions at the bottom surface of the film, *x* = −*t*_1_, can be written in following form:

(9) $$\begin{array}{l} e_{2}^{\left(1\right)}(-t_{1})=e_{3}(-t_{1})+U_{\mathrm{dr}}/l\;,\\ h_{2}^{\left(1\right)}(-t_{1})=h_{3}(-t_{1})\;. \end{array}$$

Similar expressions can be found at the top surface of the film structure, *x* = *t*_2_:

(10) $$\begin{array}{l} e_{2}^{\left(m+n+2\right)}(t_{2})=e_{4}(t_{2})+U_{\mathrm{dr}}/l\;,\\ h_{2}^{\left(m+n+2\right)}(t_{2})=h_{4}(t_{2})\;. \end{array}$$

When the field distribution is obtained, we can find the impedance, *Z*, of the multilayered film as a ratio of the applied driving voltage to the total current, *I*, flowing through the film structure:

(11) $$Z=\frac{U_{\mathrm{dr}}}{I}=\frac{U_{\mathrm{dr}}}{w\int_{-t_{1}}^{t_{2}}\sigma (x)e(x)dx}=\frac{4\pi }{cw}\times \frac{U_{\mathrm{dr}}}{h_{4}(t_{2})-h_{3}(-t_{1})}\;.$$

To describe a relative variation of the impedance, let us introduce the MI ratio, Δ*Z*/*Z*, which is given by:

(12) $$\Delta Z/Z\;(\%)=100\times [Z(H_{e})-Z(H_{0})]/Z(H_{0})\;,$$

where *H*_0_ is the value of the external field sufficient to saturate the impedance. In the further calculations, we assume that *H*_0_ = 100 Oe, which is the typical magnitude of the maximum value of the experimentally available external magnetic field [26,31].

## 3. Results

### 3.1. Influence of Multilayer Parameters on MI Response

**Figure 2:**
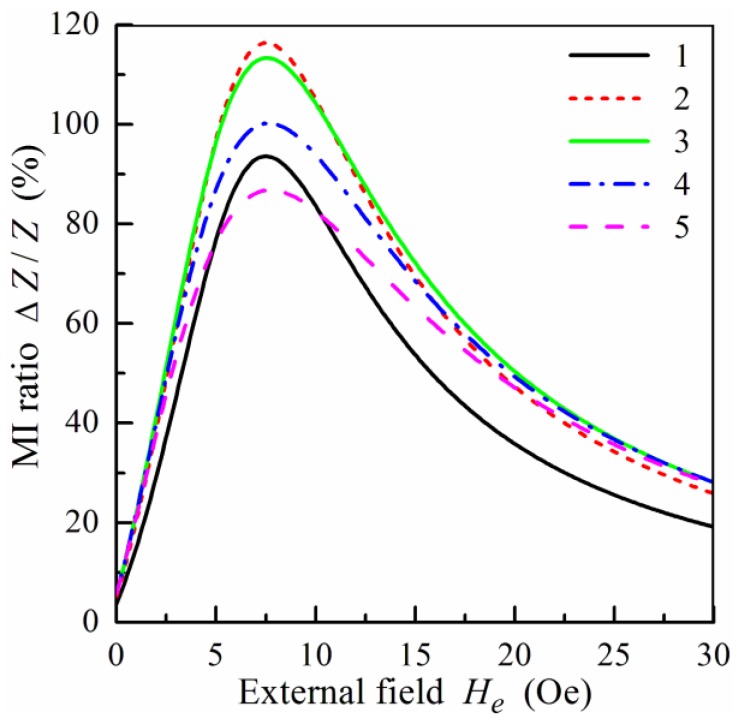
MI ratio, Δ*Z*/*Z*, as a function of the external field, *H_e_*, for different frequencies, *f*: curve 1, *f* = 25 MHz; curve 2, *f* = 50 MHz; curve 3, *f* = 100 MHz; curve 4, *f* = 150 MHz; curve 5, *f* = 200 MHz. Parameters used for calculations are *l* = 1 cm, *w* = 0.02 cm, 2*d*_0_ = 500 nm, *d*_1_ = 3 nm, *d*_2_ = 100 nm, *m* = *n* = 4, *M* = 750 G, *H_a_* = 6 Oe, *ψ* = 0.1*π*, *σ*_0_ = *σ*_1_ = 5 × 10^17^ s^−1^, *σ*_2_ = 3 × 10^16^ s^−1^, and *κ* = 0.02.

In this subsection, we analyze the results of the modeling of the field and frequency dependences of the MI in symmetric nanostructured multilayers. Let us assume that the central layer, C, and non-magnetic spacers, X, are made of the same material and, correspondingly, *σ*_0_ = *σ*_1_. The field dependence of the MI ratio for the multilayered film calculated for different frequencies is shown in Figure 2. Note that the results are presented only for the region of the positive fields and the calculated curves are symmetrical with respect to the sign of the external magnetic field, since hysteresis effects are neglected in the framework of the model. The dependence of the MI ratio on the external field shows a typical behavior with a maximum near the anisotropy field, *H_a_*. It follows from Figure 2 that the maximum values of the MI ratio are achieved within the frequency range from 50 to 100 MHz.

**Figure 3:**
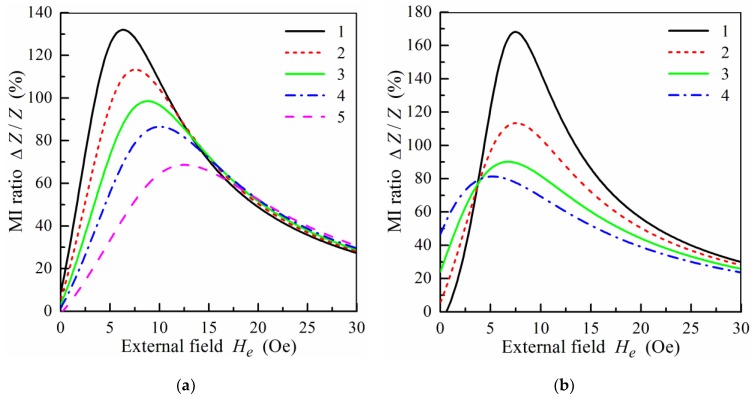
(**a**) MI ratio, Δ*Z*/*Z*, as a function of the external field, *H_e_*, at *f* = 100 MHz for *ψ* = 0.1*π* and different values of the anisotropy field, *H_a_*: curve 1, *H_a_* = 5 Oe; curve 2, *H_a_* = 6 Oe; curve 3, *H_a_* = 7 Oe; curve 4, *H_a_* = 8 Oe; curve 5, *H_a_* = 10 Oe. (**b**) Δ*Z*/*Z* ratio as a function of the external field, *H_e_*, at *f* = 100 MHz for *H_a_* = 6 Oe and different values of the anisotropy angle, *ψ*: curve 1, *ψ* = 0.05*π*; curve 2, *ψ* = 0.1*π*; curve 3, *ψ* = 0.15*π*; curve 4, *ψ* = 0.2*π*. Other parameters used for calculations are the same as in Figure 2.

Figure 3 illustrates the influence of the anisotropy field, *H_a_*, and the anisotropy axis angle, *ψ*, in the magnetic layers on the field dependence of the MI ratio. With a decrease of *H_a_*, the MI ratio increases due to a growth of the transverse permeability. At the same time, the position of the peak in the field dependence of the impedance shifts towards the zero field with a decrease of the anisotropy field (see Figure 3a). As follows, from Figure 3b, the MI ratio is very sensitive to the value of the anisotropy axis angle, *ψ*, in the ferromagnetic layers. The MI ratio drops sharply with an increase of the deviation of the anisotropy axis from the transverse direction.

Let us study the influence of the multilayer geometric parameters on the MI. For the analysis, we use the maximum MI ratio, (Δ*Z*/*Z*)_max_, which is defined as follows:

(13) $${\left(\Delta Z/Z\right)}_{\max}\;(\%)=100\times [Z_{\max}-Z(H_{0})]/Z(H_{0})\;,$$

where *Z*_max_ corresponds to the peak in the field dependence of the multilayer impedance.

**Figure 4:**
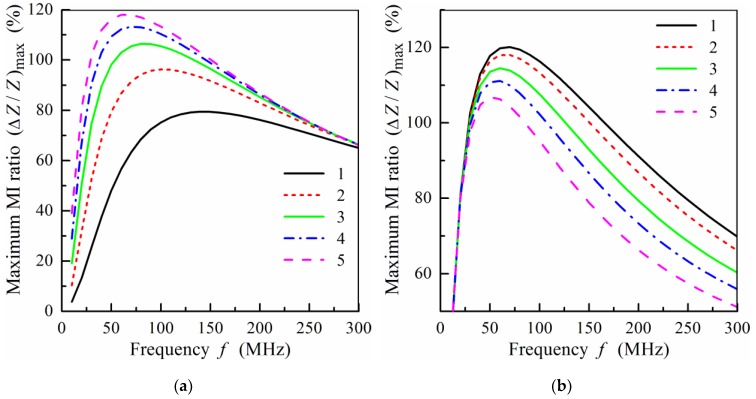
(**a**) Frequency dependence of the maximum MI ratio, (Δ*Z*/*Z*)_max_, at different values of the central layer thickness, 2*d*_0_: curve 1, 2*d*_0_ = 100 nm; curve 2, 2*d*_0_ = 200 nm; curve 3, 2*d*_0_ = 300 nm; curve 4, 2*d*_0_ = 400 nm; curve 5, 2*d*_0_ = 500 nm. (**b**) Frequency dependence of the maximum MI ratio, (Δ*Z*/*Z*)_max_, at different values of the thickness, *d*_1_, of spacers: curve 1, *d*_1_ = 2 nm; curve 2, *d*_1_ = 3 nm; curve 3, *d*_1_ = 5 nm; curve 4, *d*_1_ = 7 nm; curve 5, *d*_1_ = 10 nm. Other parameters used for calculations are the same as in Figure 2.

Figure 4a shows the frequency dependence of the maximum MI ratio, (Δ*Z*/*Z*)_max_, calculated for multilayered films with different thicknesses, 2*d*_0_, of the central layer. The value of (Δ*Z*/*Z*)_max_ decreases with the thickness of the central layer, and the peak in the frequency dependence of (Δ*Z*/*Z*)_max_ shifts towards higher frequencies with a decrease of 2*d*_0_. The results obtained are in qualitative agreement with the experimental data [24] and the results of simulation by means of the finite element method [27].

Figure 4b presents the effect of the thickness, *d*_1_, of separating layers on the frequency dependence of (Δ*Z*/*Z*)_max_. Maximal values of (Δ*Z*/*Z*)_max_ are attained at low *d*_1_, i.e., the increase of the thickness of non-magnetic spacers results in a decrease of the MI. It should be noted, however, that at low values of *d*_1_, the exchange interactions between magnetic layers appear, which can essentially influence the MI response. The critical thickness of the non-magnetic separating layer depends significantly on the properties of the magnetic layers.

**Figure 5:**
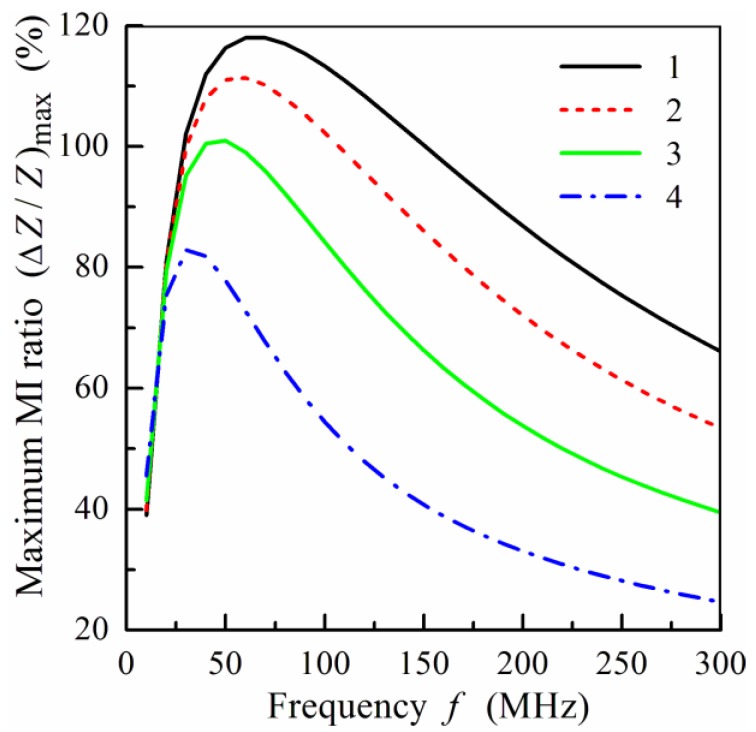
Frequency dependence of the maximum MI ratio, (Δ*Z*/*Z*)_max_, for different symmetric film structures, *m* = *n*: curve 1, *m* = 4 and *d*_2_ = 100 nm; curve 2, *m* = 9 and *d*_2_ = 50 nm; curve 3, *m* = 19 and *d*_2_ = 25 nm; curve 4, *m* = 49 and *d*_2_ = 10 nm. Other parameters used for calculations are the same as in Figure 2.

The effect of the number of magnetic layers on the frequency dependence of (Δ*Z*/*Z*)_max_ is shown in Figure 5. Note that the total thickness of the magnetic layers is constant for all films used for calculation. It follows from Figure 5 that the value of (Δ*Z*/*Z*)_max_ drops with the increase of the number of magnetic layers and with the corresponding decrease of the magnetic layer thickness.

It should be noted that the magnetic properties of the magnetic layers may change with the thickness of layers and this fact may affect significantly the MI response. In particular, an experimental study [43] showed that multilayers composed with permalloy layers of a thickness of 50 and 100 nm exhibit a similar MI ratio, whereas multilayers with thinner magnetic layers have a lower MI response. An opposite tendency was observed in another experiment [44], where it was found that the film structures with magnetic layers of a thickness of 25 nm exhibit a much higher MI ratio than films with magnetic layers with a thickness of 170 nm. This disagreement between the experimental data [44] and results of the modeling may be due to the fact that soft magnetic properties degrade in films with thick layers as a result of an approximation toward the transition into the “transcritical state” [17]. Mixed interfaces can also contribute to the balance. The volume corresponding to the interfaces is similar for multilayers with different thicknesses of magnetic layers, but the ratio between the total volume and the volume corresponding to the interfaces is different for thin and thick layers. Another contribution may come from the difference in the roughness of the interfaces corresponding to multilayers with different thicknesses of magnetic layers.

**Figure 6:**
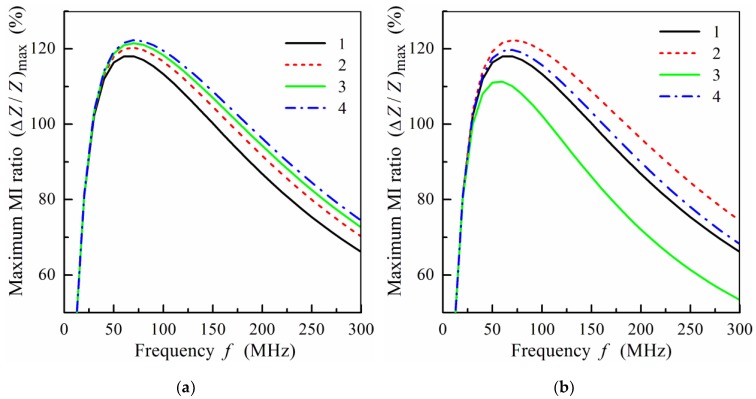
(**a**) Frequency dependence of the maximum MI ratio, (Δ*Z*/*Z*)_max_, for *m* = 4, *d*_2_ = 100 nm, and different values of the conductivity, *σ*_1_, of spacers: curve 1, *σ*_1_ = 5 × 10^17^ s^−1^; curve 2, *σ*_1_ = 2 × 10^17^ s^−1^; curve 3, *σ*_1_ = 10^17^ s^−1^; curve 4, *σ*_1_ = 5 × 10^16^ s^−1^. (**b**) Frequency dependence of the maximum MI ratio, (Δ*Z*/*Z*)_max_, at different values of the conductivity, *σ*_1_, and thickness, *d*_1_, of spacers: curve 1, *m* = 4, *d*_2_ = 100 nm, and *σ*_1_ = 5 × 10^17^ s^−1^; curve 2, *m* = 4, *d*_2_ = 100 nm, and *σ*_1_ = 5 × 10^16^ s^−1^; curve 3, *m* = 9, *d*_2_ = 50 nm, and *σ*_1_ = 5 × 10^17^ s^−1^; curve 4, *m* = 9, *d*_2_ = 50 nm, and *σ*_1_ = 5 × 10^16^ s^−1^. Other parameters used for calculations are the same as in Figure 2.

As mentioned above, materials of the central layer and non-magnetic spacers may be different. Figure 6 shows the influence of the difference in the conductivity of the central layer and spacers on the MI. It follows from Figure 6a that the value of (Δ*Z*/*Z*)_max_ increases with a decrease of the conductivity, *σ*_1_, of the separating layers. Note that the values of *σ*_1_ = 5 × 10^17^s^−1^ and *σ*_1_ = 5 × 10^16^ s^−1^ correspond approximately to the conductivity of copper and titanium. As follows from Figure 6b, replacing copper with titanium has a more significant effect on the MI response, when the thickness of the spacers decreases.

### 3.2. MI in Non-Symmetric Nanostructured Multilayers

**Figure 7:**
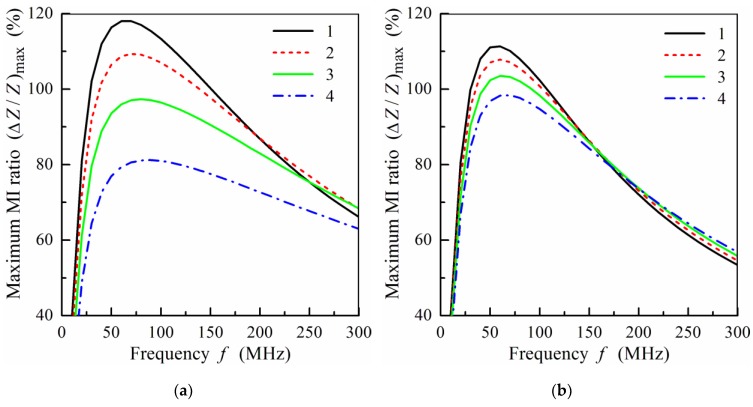
(**a**) Frequency dependence of the maximum MI ratio, (Δ*Z*/*Z*)_max_, at *m* = 4, *d*_2_ = 100 nm, and different values of *n*: curve 1, *n* = 4; curve 2, *n* = 3; curve 3, *n* = 2; curve 4, *n* = 1. (**b**) Frequency dependence of the maximum MI ratio, (Δ*Z*/*Z*)_max_, at *m* = 9, *d*_2_ = 50nm, and different values of *n*: curve 1, *n* = 9; curve 2, *n* = 8; curve 3, *n* = 7; curve 4, *n* = 6. Other parameters used for calculations are the same as in Figure 2.

Let us now study the MI effect in non-symmetric multilayered structures. The frequency dependence of the maximum MI ratio, (Δ*Z*/*Z*)_max_, calculated for film structures with different numbers of layers is shown in Figure 7. It is assumed that the properties of the magnetic layers are the same for the symmetric, *n* = *m*, and non-symmetric films, *n* < *m*. It follows from Figure 7a that the value of (Δ*Z*/*Z*)_max_ decreases with the number of layers, *n*. The frequency of the peak in (Δ*Z*/*Z*)_max_ increases with the growth of asymmetry between the top and bottom layers. Within the frequency range, *f* > 250 MHz, non-symmetric multilayered films exhibit a higher MI effect.

Figure 7b presents the results of calculations of (Δ*Z*/*Z*)_max_ for the films with thinner magnetic layers. In this case, the decrease in (Δ*Z*/*Z*)_max_ for the non-symmetric films is less pronounced in comparison with the symmetric film structure, *n* = 9. At the frequencies of the order of 150 MHz and higher the symmetric and non-symmetric films show very similar magnitudes of (Δ*Z*/*Z*)_max_ (Figure 7b). For practical purposes, a lower working frequency may have higher importance in comparison with the maximum effect value. From this point of view, the result corresponding to *n* = 9 is very interesting as the (Δ*Z*/*Z*)_max_ peak appears at a lower frequency in comparison with the *n* = 4 curve (Figure 7a).

## 4. Discussion

The aim of the work was to develop a model and theoretical analysis of MI in multilayer films with nanostructured magnetic layers. Up to now, such a model description was absent in the research literature. The results of modeling are in qualitative agreement with experimental studies of the MI in non-symmetric multilayers [31,32,44]. However, in the experiments, the change in the frequency of the peak in (Δ*Z*/*Z*)_max_ is more pronounced, when the difference in the thickness of the top and bottom layers increases. Moreover, it was found that the frequency dependence of (Δ*Z*/*Z*)_max_ differs significantly for the multilayers with odd and even numbers, *n*, in the bottom layer [32]. These facts clearly indicate that magnetostatic interactions between magnetic layers affect significantly the MI in non-symmetric multilayers. In fact, in all previous designs of the multilayers, the main priority was given to the evaluation of the coercivity of single-layer or three-layered structures. For example, the interaction between two magnetic layers separated by a weakly magnetic layer for Fe_19_Ni_81_/Cr/Fe_19_Ni_81_ and Fe_15_Co_20_Ni_65_/Cr/Fe_15_Co_20_Ni_65_ was studied [45]. The experimental data on the coercivity, domain structure parameters, and microstructure as well as the theoretical estimates showed that an increase of the thickness of the Cr can lead to a replacement of the exchange interaction between the ferromagnetic layers by the magnetostatic interaction. In its turn, the effectiveness of the magnetostatic interaction can be governed by surface defects and the layer magnetization ripple structure [45]. These conclusions correspond to the simplest symmetric structure, F/X/F, even without a central conductor, but the results for F/X/F/X/F, F/X/F/X/F/X/F, etc. are absent in the literature.

One of the weak points in a comparison of the magnetic properties of multilayered structures is a well known dependence of the properties of thin films on the preparation conditions and even particular equipment [46,47]. In combination with the strong dependence of the interaction between two magnetic layers on the thickness of the non-magnetic spacer, varying at nanoscale, the comparison becomes a very difficult task. With respect to MI multilayers, the experimental data on the adjustment thickness of the spacers are very limited. The microstructure and magnetic properties of FeNi films and FeNi (170 nm)/M/FeNi (170 nm) (M = Co, Fe, Gd, Gd-Co) multilayers were studied in [48]. In contrast to the Co and Fe spacers, Gd and Gd-Co magnetic spacers improved the softness of the FeNi/X/FeNi multilayers. The MI responses were also measured, and the highest MI variation was observed for the [FeNi/Gd (2 nm)]_2_/FeNi case. The thickness for the minimum of coercivity in the case of the Gd spacer was 3 ± 1 nm, which is almost the accuracy limit for the sputtering technique. Although the MI measurements were performed for [FeNi/Gd (2 nm)]_2_/FeNi multilayers, the behavior of the system for different spacer thicknesses in the cases of different numbers of magnetic layers was not studied systematically. All this means that we still need to make experimental efforts in order to improve the phenomenological basis for the next step of the theoretical development of the problem of MI multilayers.

In order to understand the role of the magnetostatic interactions, a more systematic investigation is required. In the framework of the model proposed, the magnetostatic interactions can be taken into account by introducing an additional effective field acting on the bottom layer of the film structure. Although this approach simplifies the real field distribution, it allows one to describe qualitatively the influence of the magnetostatic field on the MI in non-symmetric multilayers.

We would like to now return to the concept of the magnetic MI biosensor. Why is the problem of symmetric or non-symmetric MI structures so important in this particular case? There are two main different types of biomedical requests: Analysis of electric and magnetic properties of living systems, reflecting their functionality, and analysis of specific properties of the biocomponents. In the present work, we refer to both the first and the second kind. The first case was already demonstrated as useful devices for biomagnetic level magnetic field recording (magnetocardiogram or magnetoencefalogramm) [49] or a very first attempt to use MI detectors for diagnostics of vascular problems near stenosis [50].

**Figure 8:**
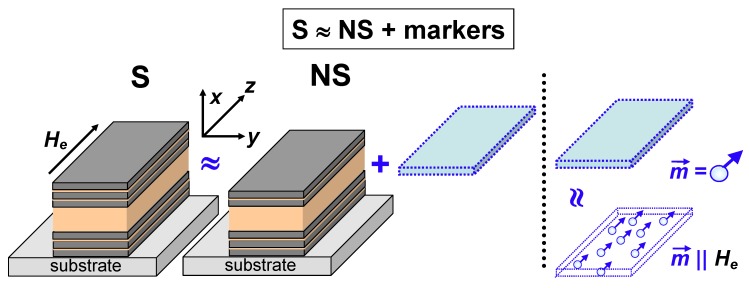
Schematic representation of the symmetric (S) and non-symmetric (NS) MI multilayered sensitive elements. In a model case (to mimic the layer of randomly distributed magnetic markers), the top multilayer can be substituted by a set of superparamagnetic spherical markers with the same individual moments, $\vec{m}$, oriented in the direction of the applied field, *H_e_*.

The second kind are magnetic marker detecting compact analytical devices [29,30,51]. As the main principle of magnetic marker detection is an evaluation of the sum of the stray fields of all magnetizable markers [52], their conjunction can be viewed as an additional layer with particular properties (Figure 8). Magnetic markers for biomagnetic detection are spherical superparamagnetic nanoparticles or polymer composites containing spherical superparamagnetic nanoparticles, usually biocopatible iron oxides [53,54]. In the ideal case, they are all identical and carry the same magnetic moments, $\vec{m}$, in a certain applied magnetic field. External field and magnetic moments of individual markers are parallel to each other and therefore each marker creates stray fields. The measured difference between the sensor output in the absence of the magnetic markers and in their presence allows (in the calibrated system) a calculation of the amount of magnetic markers and therefore the biocomponents of interest [55,56]. One therefore treats the study of the comparison of symmetric and non-symmetric cases for MI multilayers as a model approach for improving the MI biodetector sensitivity.

A theoretical study of the MI in symmetric and non-symmetric nanostructured multilayers can be useful for the development of planar detectors of very low magnetic fields (of the order of biomagnetic responses) of both types described above. Of course, proper comparison of the experimental results and specially designed MI multilayers with nanostructured magnetic layers is desirable and we are now in the process of obtaining the set of required multilayered samples for comparison. 

## 5. Conclusions

The MI effect in symmetric and non-symmetric multilayers with nanostructured magnetic layers was studied theoretically in order to analyze the influence of the multilayer parameters on the field and frequency dependences of the MI response. The proposed approach consists of a calculation of the field distribution within the multilayered film by means of a simultaneous solution of lineralizied Maxwell equations and the Landau–Lifshitz equation. The model developed allows one to explain qualitatively the main features of the MI effect in multilayers. It can be useful for optimization of the MI film parameters. It might also be useful as a model case for the development of MI magnetic biosensors for magnetic marker detection.
